# Cepharanthine suppresses *APC*-mutant colorectal cancers by down-regulating the expression of β-catenin

**DOI:** 10.1007/s13659-024-00443-1

**Published:** 2024-02-29

**Authors:** Guifeng Su, Dan Wang, Qianqing Yang, Lingmei Kong, Xiaoman Ju, Qihong Yang, Yiying Zhu, Shaohua Zhang, Yan Li

**Affiliations:** 1 State Key Laboratory of Phytochemistry and Plant Resources in West China, Kunming Institute of Botany, Chinese Academy of Sciences, Kunming, 650201 People’s Republic of China; 2 Key Laboratory of Medicinal Chemistry for Natural Resource, Ministry of Education, Yunnan Key Laboratory of Research and Development for Natural Products, School of Pharmacy, Yunnan University, Kunming, 650500 People’s Republic of China; 3 University of Chinese Academy of Sciences, Beijing, 100049 People’s Republic of China

**Keywords:** Colorectal cancer, Wnt/β-catenin, *APC*, CEP, Transcriptional inhibitor

## Abstract

The aberrant activation of the Wnt/β-catenin signaling pathway is closely associated with the development of various carcinomas, especially colorectal cancers (CRCs), where adenomatous colorectal polyposis (APC) mutations are the most frequently observed, which limits the anti-tumor efficiency of inhibitors targeting the upstream of Wnt/β-catenin pathway. The anti-tumor activity of the naturally occurring alkaloid cepharanthine (CEP) extracted from the plant *Stephania cepharantha *Hayata has been reported in various types of tumors. We previously observed that its derivatives inhibited the Wnt/β-catenin signaling in liver cancer; however, the specific mechanism remains unknown. In this study, we confirmed CEP can effectively inhibit *APC*-mutant CRC cell lines (SW480, SW620, LoVo) through disturbing of the Wnt/β-catenin signaling and elucidated the underlying mechanisms. Here, we demonstrate that CEP attenuates the Wnt/β-catenin signaling by decreasing the β-catenin, subsequently impeding the proliferation of *APC*-mutant CRCs. Moreover, CEP induced β-catenin transcription inhibition rather than the instability of β-catenin protein and mRNA contributes to reduction of β-catenin. Taken together, our findings identify CEP as the first β-catenin transcriptional inhibitor in the modulation of Wnt/β-catenin signaling and indicate CEP as a potential therapeutic option for the treatment of *APC*-mutated CRCs.

**Graphical Abstract:**

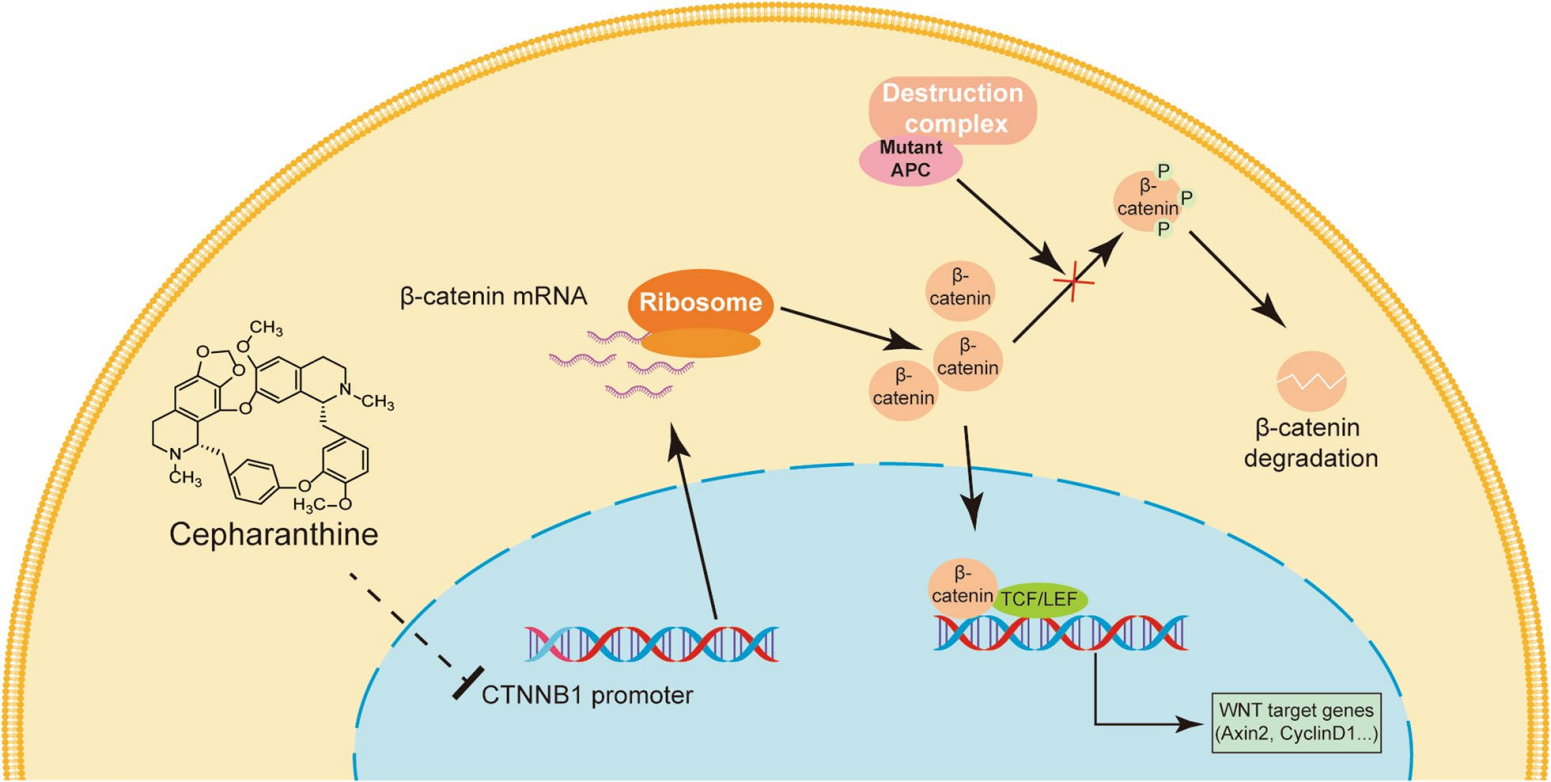

## Introduction

Colorectal cancer (CRC) is a frequently occurring cancer in the digestive system, known for its high mortality and incidence rates globally [1]. According to the tumor location, CRC can be further divided into colon cancer (CC) and rectal cancer (RC). Its incidence continues to rise annually, particularly among younger individuals, thereby presenting a significant health concern for the population [2]. Despite significant advancements in the clinical treatment of CRC through surgical resection combined with chemotherapy and radiotherapy, the overall prognosis for CRC patients, especially for patients diagnosed at advanced stages, remains unfavorable [3]. The current chemotherapy drugs for CRCs, such as fluoropyrimidine, oxaliplatin, and capecitabine, are still limited by issues of resistance and toxicity, which hinder the effectiveness of the therapy [4]. Therefore, novel and more effective potential therapeutic drugs are in urgent for the treatment of CRCs.

Wnt/β-catenin signaling pathway is an evolutionarily conserved cascade involved maintenance of stem cells, tissue homoeostasis and embryonic development [5]. The dysregulation of the Wnt pathway is intricately linked to a variety of diseases, including degenerative diseases, metabolic diseases, cardiovascular diseases, and particularly cancer [6]. Dyregulation of the Wnt/β-catenin cascade expedites cancer cell proliferation, facilitates tumor invasion, and induces drug resistance, particularly in CRCs [7]. In CRCs, the most commonly mutated proteins are *APC* and β-catenin, and *APC* mutations are particularly prevalent and can be found in up to 80% of CRCs [8]. Both inactive *APC* mutation and activating β-catenin mutation contribute to the nuclear translocation of β-catenin and subsequent initiation and progression of CRCs. In recent years, the canonical Wnt/β-catenin signaling has gained significant attention as a druggable target for CRCs. Of these, Acyl hydrazones (M-110, OICR62321H7) destabilize β-catenin by chelating iron, which is a critical requirement for the Wnt signaling pathway [7, 9]. JW67 and JW74 reduce activated β-catenin by promoting β-catenin phosphorylation and degradation, and the small molecule ICG-001 effectively interrupts β-catenin/TCF transcription by specifically targeting the cyclic AMP response element binding protein (CREB) [10, 11]. Currently, preclinical studies and clinical trials are in progress to evaluate the effectiveness of inhibitors targeting the Wnt/β-catenin cascade in the treatment of CRCs.

Cepharanthine (CEP) is an alkaloid isolated from the plant *Stephania cepharantha *Hayata [12], utilized in Japan to treat radiotherapy-induced leukopenia [13], alopecia areata [14] and snakebite [15] for over 70 years, with no significant adverse effects reported [16]. Various anti-cancer mechanisms of CEP have been reported, including the NF-κb signaling pathway inhibition [17], JNK1/2 and AKT activation to induce chromatin condensation and nuclear fragmentation [18], increase of p21Waf1/Cip1 and decrease of cyclin A and Bcl-2, induction of ROS production [19], AMPK activation induced autophagy associated cell death [20], and reduction in STAT3 [21]. As previously reported in our work, CEP can effectively inhibit the Wnt/β-catenin signaling in liver cancer cells [22], with the precise mechanism by which CEP inhibits the Wnt/β-catenin pathway remains to be fully understood. In this study, the Wnt/β-catenin signaling inhibition and subsequent growth suppression of CEP in CRC cell lines with *APC* mutations were detected. This study will offer valuable insights into the potential clinical application of CEP in Wnt/β-catenin signaling driven CRCs.

## Results

### CEP inhibits proliferation of *APC*-mutant CRCs

**Fig. 1:**
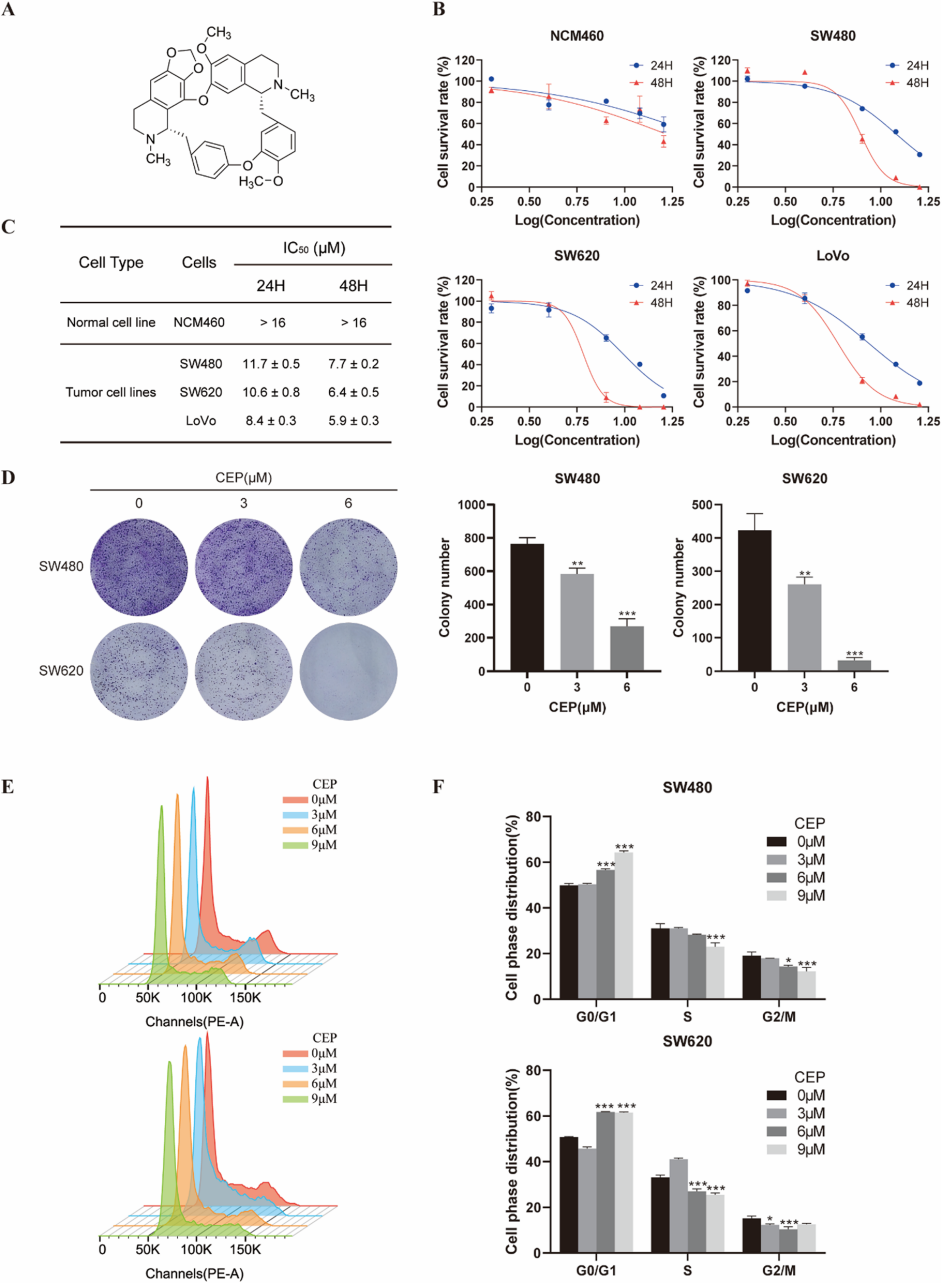
CEP suppresses the growth of *APC*-mutant CRCs. **A** Chemical structure of CEP. **B** Cell viability of NCM460, SW480, SW620 and LoVo cells exposed to CEP (0–16 μM) for 24, 48 h was determined by MTS assay. **C** IC_50_ concentrations were estimated based on dose–response curves using the GraphPad software. **D** Representative images of the cell clone formation assay for SW480 and SW620 cells exposed to CEP at indicated concentrations in plates for 2 weeks. **E** Flow cytometry analysis of cell cycle distribution in *APC*-mutant CRCs treated with CEP. **F** Quantification data of cells distributed at each stage of cell cycle

To investigate the growth inhibitory effect of CEP (Fig. 1A) on *APC*-mutant CRC cells, SW480, LoVo and SW620 cells with *APC* mutations were treated with CEP for 24 or 48 h. Results from MTS assay demonstrate that both dose and time dependent cell viability loss was observed in CEP exposed CRC cells, with IC_50_ values of CEP following 48 h treatment ranging from 5.9 μM to 11.7 μM (Fig. 1B, C), while the human colon mucosal epithelial cell line NCM460 cells exhibited relative tolerance to CEP treatment, with the IC_50_ value exceeding 16 μM (Fig. 1B, C). Furthermore, the number of tumor clones formed in SW480 and SW620 cells significantly decreased in the presence of CEP, suggesting CEP effectively inhibits the proliferation of both CRC cell lines (Fig. 1D). Moreover, the proportion of cells in the G1/G0 phase were increased after the treatment of CEP (Fig. 1E, F), demonstrating that CEP induces cell cycle arrest in G1/G0 phase in *APC*-mutant CRCs. Together, these results suggest that CEP selectively inhibits the growth and proliferation of *APC*-mutant CRCs.

### CEP attenuates transcriptional activity of β‐catenin/TCF4 in *APC*-mutant CRCs

**Fig. 2:**
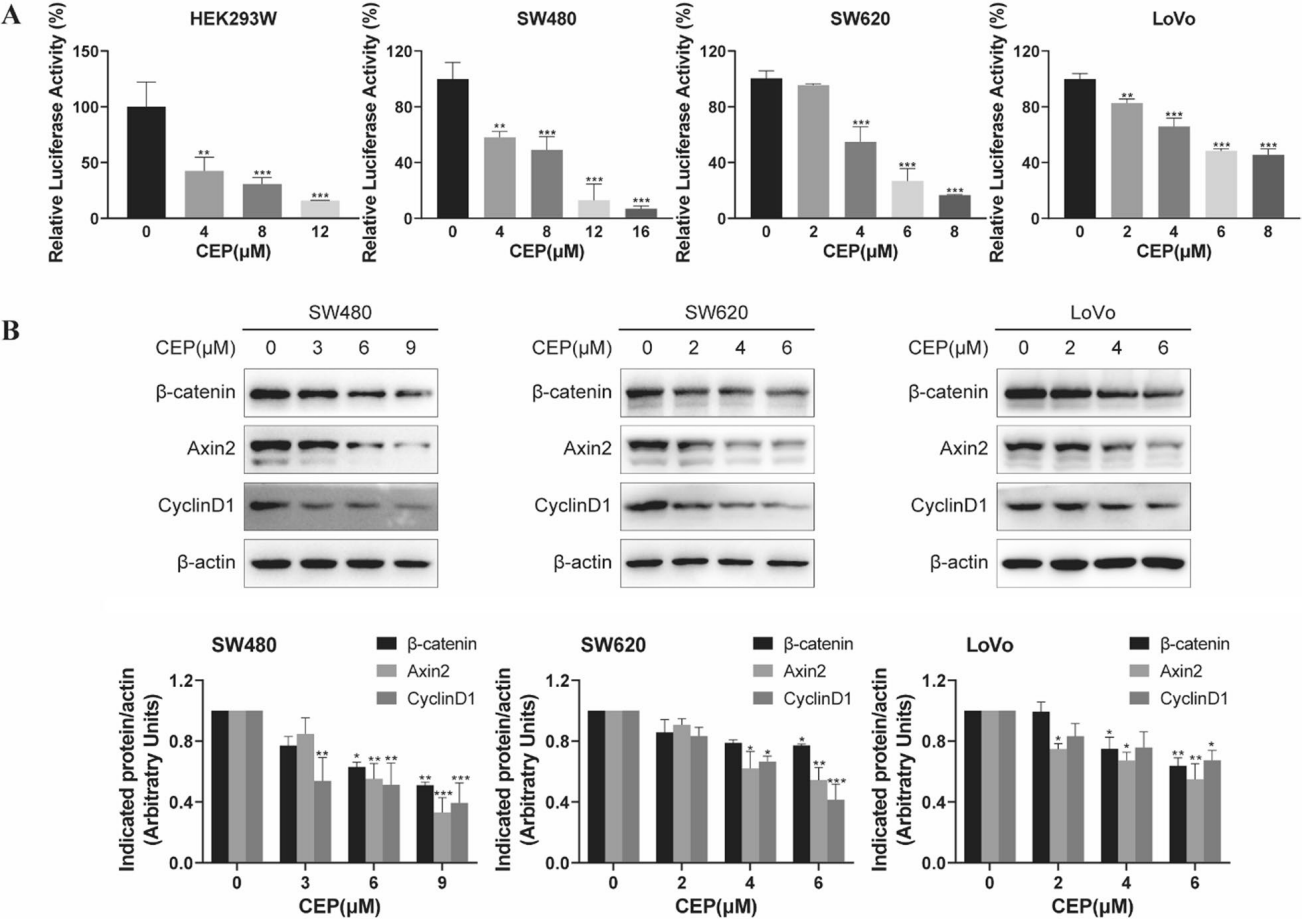
Inhibition of CEP on the transcriptional activity of β‐catenin/TCF4 in *APC*-mutant CRCs. **A** *APC*-mutant CRCs were transfected with TOPflash luciferase reporter and Renilla plamids and then exposed to increasing concentrations of CEP, with HEK293W cells treated with indicated concentrations CEP directly for 24 h, followed by the measurement of the luciferase activity. **B** Western blot analysis of proteins of β-catenin, Axin2 and CyclinD1 in *APC*-mutant CRCs. Quantification of the protein expression was performed using ImageJ software

*APC* mutation in CRCs leads to the hyperactivaiton of the Wnt/β-catenin signaling and upregulation of the target genes [23]. Consequently, we investigated whether CEP could effectively inhibit Wnt/β-catenin signaling. Firstly, the dual-luciferase reporter assay was performed with HEK293W cells constructed by stably co-transfecting HEK293 cell line with Wnt3a, Renilla and SuperTOPFlash plasmid [24]. As demonstrated in Fig. 2A, CEP treatment caused a dose-dependent decrease of the SuperTOPFlash luciferase activity. Moreover, the transcriptional inhibitory activity of β-catenin/TCF in *APC* mutant CRC cell lines with hyperactivated Wnt/β-catenin signaling was further investigated. When the concentration of CEP was increased, the activity of SuperTOPFlash luciferase in *APC*-mutated CRC cell lines (SW480, LoVo, SW620) was down-regulated in a concentration-dependent manner (Fig. 2A). The protein level of the key regulator of Wnt signaling pathway β-catenin, as well as the direct target genes of Wnt signaling pathway including Axin2, CyclinD1 were also analyzed by western blotting. β-catenin, cyclinD1 and Axin2 expression levels were significantly reduced when these cells used were treated with CEP for 48 h (Fig. 2C).

### CEP decreases the levels of nuclear β‐catenin in *APC*-mutant CRCs

**Fig. 3:**
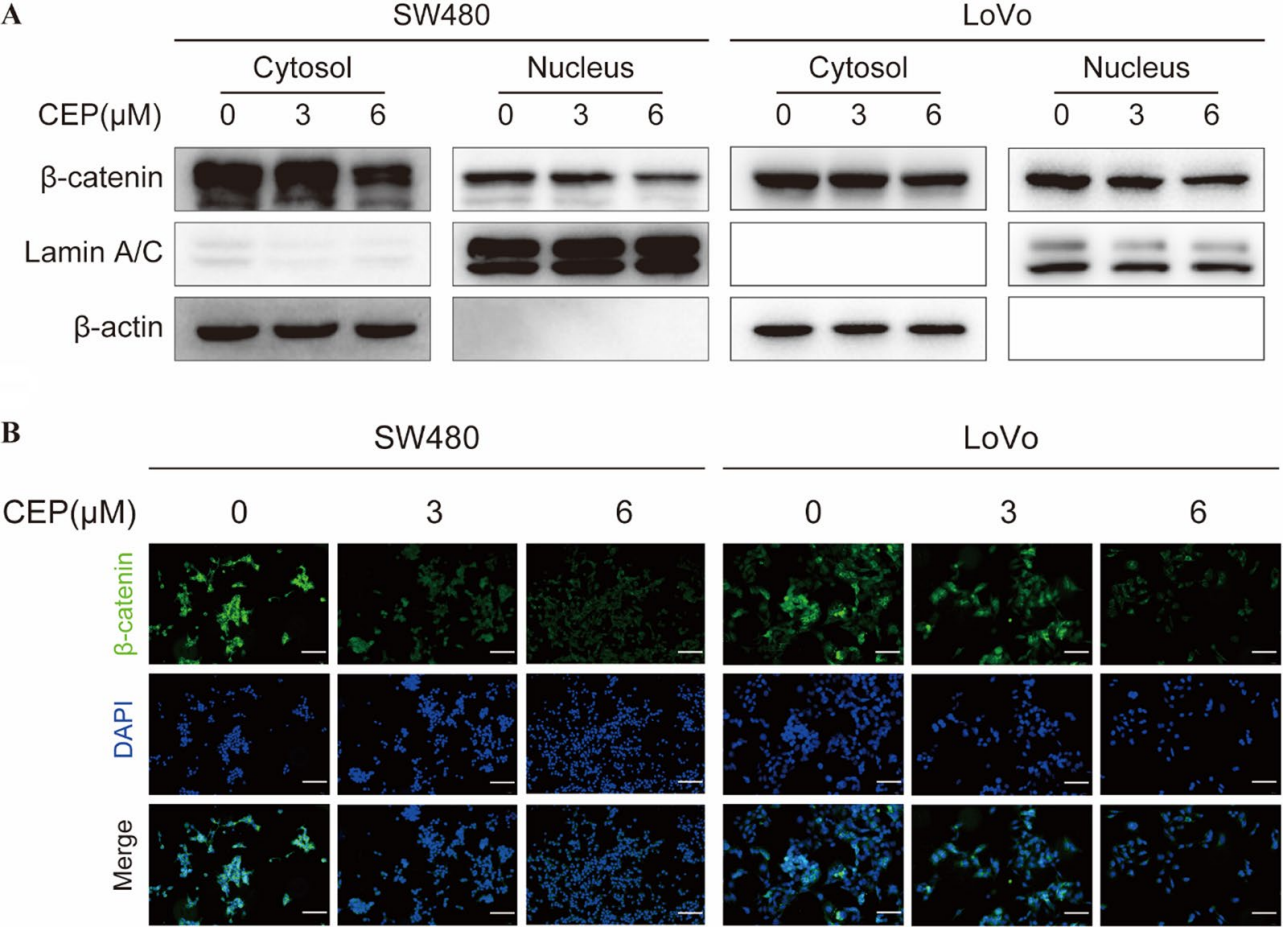
CEP decreases the nuclear β‐catenin in *APC*-mutant CRCs. **A** The Western blotting was performed to detect the β-catenin protein in cytosol and nucleus with CEP treated cells. The cytoplasm marker applied was β-actin, whereas the nucleus marker used was Lamin A/C. **B** Distribution analysis of β-catenin (green) and nucleus (blue) in SW480 and LoVo cells with Immunofluorescence analysis. Scale bar, 100 µM

*APC* mutation induced insufficient degradation of β-catenin results in excessive nucleus accumulation of β-catenin, complexing with the transcription factors TCF and LEF to initiate transcription [23]. As the total β-catenin was found to be downregulated upon the CEP treatment as evidenced by western blot analysis (Fig. 2B), we subsequently investigate alterations of β-catenin levels in the nucleus. The cytoplasmic and nuclear β-catenin were separated and analyzed. The results demonstrated both the nuclear and cytoplasmic β-catenin were decreased upon the treatment of CEP in SW480 and LoVo cells (Fig. 3A), which were further confirmed by the immunofluorescence assay (Fig. 3B).

### CEP inhibition of *APC*-mutant CRC cells proliferation is partially mediated by β-catenin

**Fig. 4:**
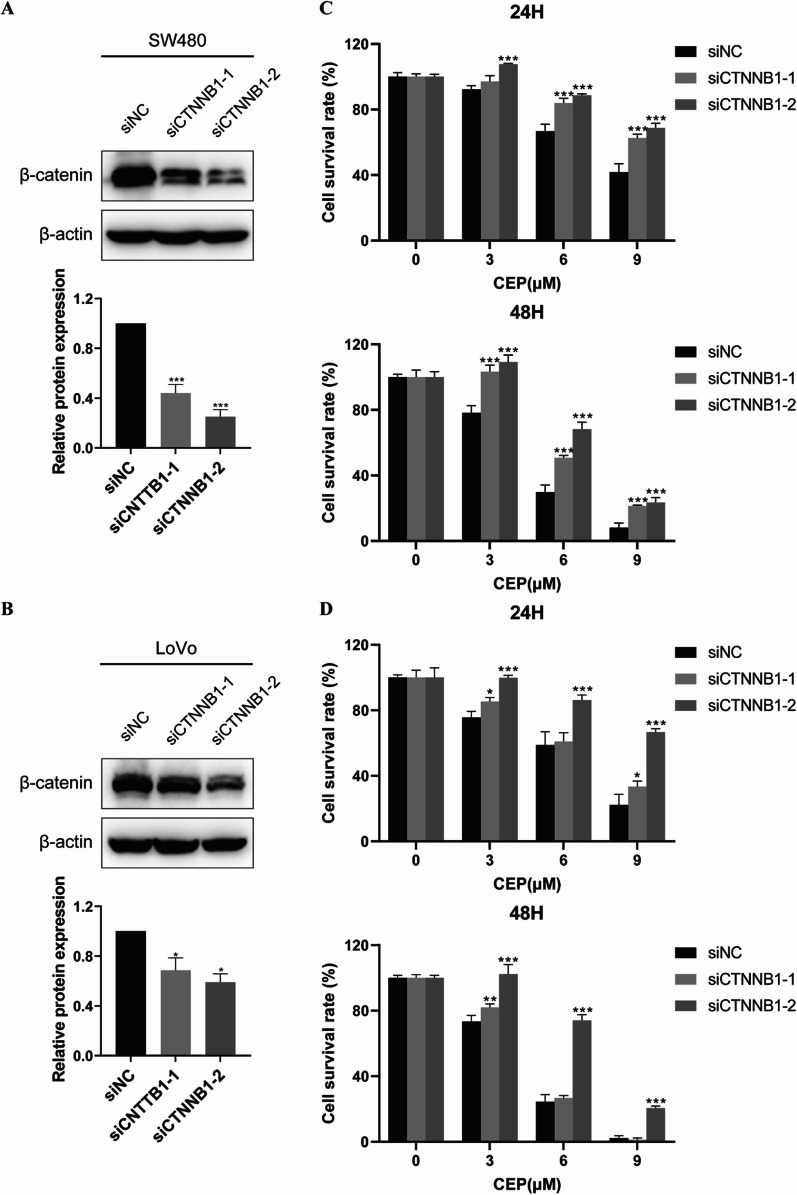
β-catenin mediates inhibition of the proliferation of *APC*-mutant CRCs by CEP. **A**, **B** β-catenin was measured by Western blotting after *CTNNB1* siRNA infection in SW480 and LoVo cells. **C**, **D** MTS assay was conducted in SW480 and LoVo cells transfected with siRNAs targeting *CTNNB1*. Relative cell viability was normalized to the CEP-free group

Given the Wnt/β-catenin signaling on the onset and progression of CRCs [25], and the inhibitory effect of CEP on this pathway, it is imperative to determine whether the Wnt/β-catenin signaling contributes to CEP induced cytotoxicity. Thus, cell viability was detected in *APC*-mutated CRC cell lines (SW480 and LoVo) transfected with siRNA targeting β-catenin (Fig. 4A, B). Compared to the negative control siRNA transfection group, the growth inhibition of *APC*-mutated CRC cells by CEP was obviously alleviated under the knockdown of β-catenin in both SW480 and LoVo cells (Fig. 4C, D). These data imply that β-catenin is involved in CEP mediated growth suppression.

### CEP diminishes β-catenin levels by inhibiting the protein synthesis

**Fig. 5:**
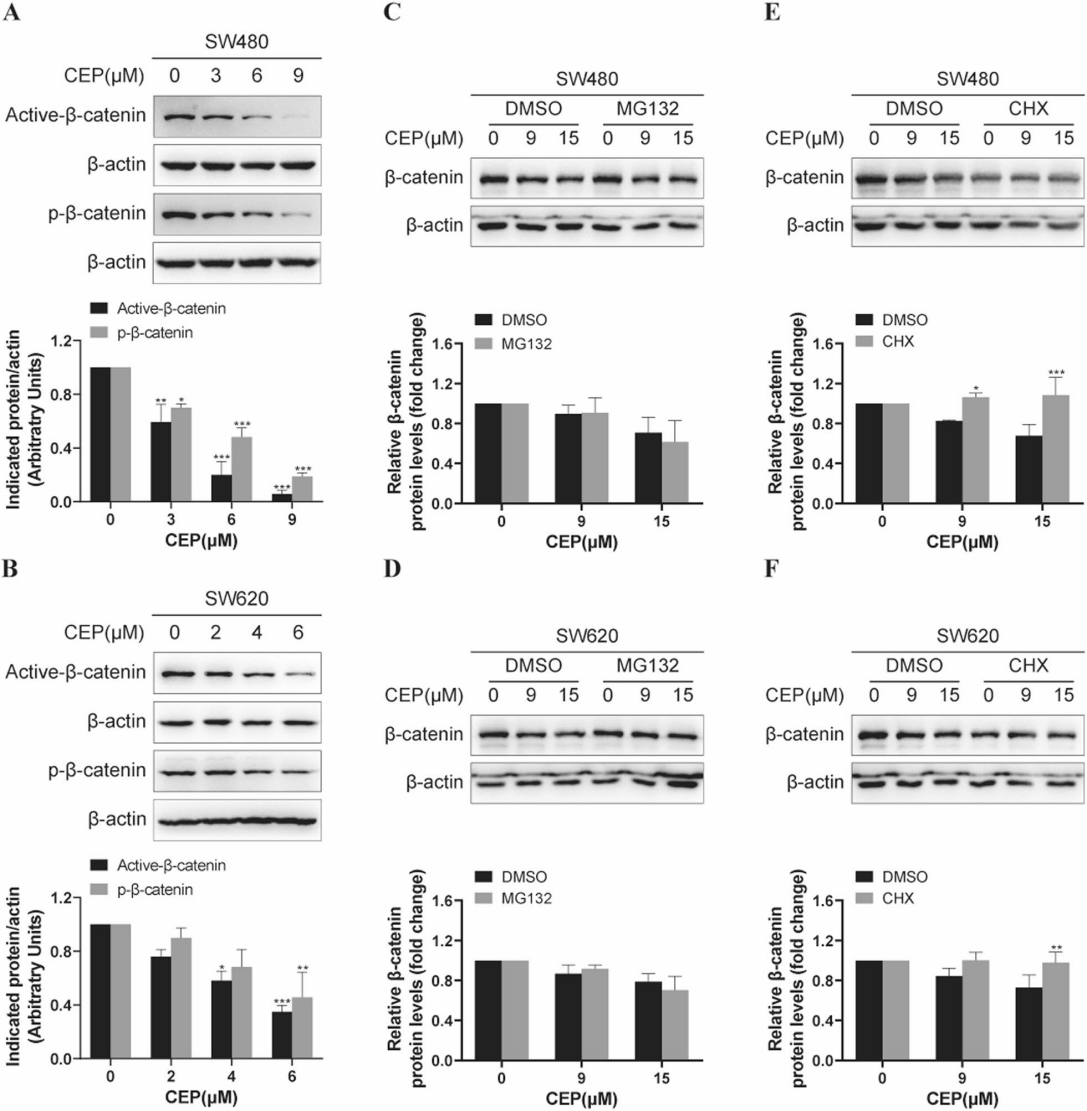
CEP reduces β-catenin protein synthesis. **A**, **B** Western blotting was applied to detect p-β-catenin, active β-catenin in SW480 and SW620 cells exposed to CEP for 48 h. **C**, **D** SW480, SW620 cells were subjected to designated dosages of CEP with or without 20 μM MG132 for 16 h, and then analyzed using western blotting. **E**, **F** Representative Western blot and quantification of β-catenin within cell extracts of CRC (SW480, SW620) cells pretreated with or without CHX (100 μg/ml) for 2 h followed by 12 h indicated concentrations of CEP exposure. The quantification of β-catenin protein expression was performed using ImageJ software. The Western blot results were quantified and standardized using β-actin as a reference

As our data indicate that CEP can facilitate the reduction of β-catenin, we next investigate the underlying mechanisms. Under normal circumstances, β-catenin undergoes phosphorylation and poly-ubiquitination by a multiprotein “destruction complex” (includes the scaffolding proteins Axin, adenomatous polyposis coli (APC), the protein kinases glycogen synthase kinase 3 (GSK3), casein kinase I (CKI), protein phosphatase 2A (PP2A), and the E3-ubiquitin ligase β-TrCP), followed by degradation through the proteasome pathway [26]. Hence, we assessed the phosphorylation states of β-catenin in SW480 and SW620 cells following treatment with gradient concentrations of CEP. Figure 5A and B demonstrated CEP led to the downregulation of the presumably transcriptionally active form of β-catenin, characterized by its unphosphorylated N-terminus. Nevertheless, the phosphorylated β-catenin is also diminished. These observations imply that CEP reduces β-catenin protein levels independently of phosphorylation promotion. Given that phosphorylated β-catenin is subject to rapid polyubiquitination and subsequent proteasomal degradation, we employed MG132 to inhibit proteasome-mediated degradation and found that treatment with MG132 hardly changed CEP induced β-catenin reduction (Fig. 5C, D), confirming that CEP does not act through the promotion of β-catenin protein degradation. Next, we investigated whether inhibiting protein synthesis with CHX affects CEP capacity to facilitate the reduction of β-catenin protein levels. Once protein synthesis is blocked by CHX, CEP failed to further downregulate β-catenin protein levels (Fig. 5E, F). Collectively, these results suggest that CEP reduces β-catenin by blocking protein synthesis, rather than influencing β-catenin protein stability.

### CEP decreases β-catenin transcription without changing the stability of the mRNA

**Fig. 6:**
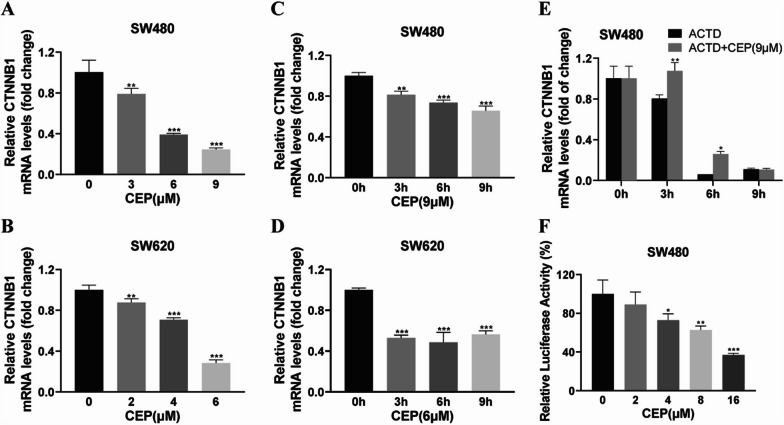
CEP decreases the transcription of β-catenin. **A**–**D** SW480 and SW620 cells administrated with CEP for indicated doses and times were subjected to RT-PCR assay, and GAPDH was used to normalize the relative expression level. **E** The mRNA levels of β-catenin were analyzed in SW480 cells exposed to 9 μM CEP for different times in the presence of ACTD. **F** SW480 cells were treated with given doses of CEP for 24 h, the luciferase activity of the *CTNNB1* promoter was determined by a luciferase reporter assay

Given that CEP does not influence the stability of β-catenin, we subsequently investigated whether CEP challenged the mRNA of β-catenin. Demonstrated in Fig. 6A–D, CEP obviously decreased β-catenin mRNA. We further investigated the mechanism underlying the inhibition of CEP on β-catenin mRNA when the mRNA transcription was inhibited by actinomycin D (ACTD). We observed that, in the presence of ACTD, the reduction of β-catenin mRNA was not enhanced with CEP treatment (Fig. 6E), suggesting that CEP does not influence the stability of β-catenin mRNA. To further investigate whether CEP downregulates β-catenin mRNA transcription, we constructed pGL3-β-catenin luciferase reporter and detected the activity to explore the impact of CEP on β-catenin transcription. The data suggested that CEP administration at varying concentrations reduced the relative luciferase activity of pGL3-β-catenin (Fig. 6F), suggesting that CEP induced the decrease in β-catenin mRNA levels through transcriptional inhibition. Taken together, CEP decreases β-catenin through transcriptional inhibition.

## Discussion and conclusion

Dysregulation of the Wnt/β-catenin pathway is intricately linked to the initiation and progression of tumors [27, 28]. Mutations in this pathway can lead to tumor metastasis, resistance to drugs, and tumor recurrence in patients undergoing chemotherapy and radiotherapy [29–31]. Therefore, small molecule compounds have been identified to target various components within the Wnt/β-catenin signaling pathway [32–36]. Despite the β-catenin-binding inhibitors have been reported, none have progressed beyond preclinical studies [37, 38].

Other strategies for downregulating the Wnt/β-catenin signaling involve β-catenin mRNA instability to specifically inhibit its translation [39, 40]. Many reports have focused on retarding the growth of preclinical tumor models by introduction of siRNA targeting β-catenin [40, 41]. It has been observed that tumor growth promptly resumes upon cessation of β-catenin silencing, highlighting the need for sustained inhibition of β-catenin [42]. However, current systemic delivery methods for siRNA still face challenges, including precise tumor targeting, limited stability, and safety [43], which could just be overcome by small molecule drugs. In this study, we identified CEP as the first β-catenin transcriptional inhibitor, with Wnt/β-catenin signaling strickingly suppressed. AS a natural leukocyte proliferative drug [13], CEP was found here for the first time to decline the growth of *APC*-mutated CRCs by reducing β-catenin. Mechanistically, CEP reduces β-catenin not through the classical pathway, which involves the phosphorylation, ubiquitination and proteasomal degradation of β-catenin, whereas by means of reducing the mRNA level of β-catenin. However, this compound does not influence mRNA stability, but reduces the β-catenin transcription. By the way, it is evident from Fig. 6E that CEP has an inclination to prevent mRNA turnover of *CTNNB1* within a brief timeframe. While this does not affect the establishment of the conclusion that CEP does not promote mRNA degradation but rather inhibits transcription, this intriguing phenomenon still warrants further investigation. β-catenin mRNA silence has been shown sufficient to inhibit tumors with intrinsic resistance or acquired resistance to trametinib [44]. In vivo, the growth of colorectal cancers, melanoma, and hepatocellular carcinoma was effectively suppressed by tumor-specific nanoparticles that contained RNAi targeting β-catenin, combined with the MEK inhibitor trametinib approved by the FDA. CEP, a small molecule with advantage of oral availability, can serve as a replacement for RNAi drugs targeting β-catenin.

It is established that CRCs are driven by mutations inactivating *APC* or activating β-catenin [45], with *APC* mutations being particularly prevalent, detected in 80% of CRC patients [8, 46]. The mutated inactivation of *APC* resulted hyperactivation of the Wnt/β-catenin signaling by disrupting the assembly of multiprotein “destruction complex” [26, 47] drives the progression of CRCs, enhances tumor cell stemness, and induces drug resistance and tumor recurrence [7, 29]. Due to this, inhibitors targeting upstream components of β-catenin [48], such as PORCN inhibitors, Wnt ligand antagonists, Frizzled receptor antagonists, FZD10 antagonists, and Tankyrase inhibitors, exhibit limited therapeutic efficacy against the majority of CRCs. However, inhibitors that specifically target β-catenin or its interactions with transcription factors and coactivators, including β-Catenin and TCF complex, β-Catenin and CBP complex, and β-Catenin and BCL9 complex, demonstrate improved therapeutic. Our study has shown that CEP can selectively inhibits β-catenin transcription and reduces its protein levels, overcoming the challenge of hyperactivation of Wnt/β-catenin signaling with *APC* mutations in CRCs.

CEP, as a clinical drug, which has received approval for the treatment of various acute and chronic diseases over a span of 70 years in Japan, has a superior safety profile exhibiting minimal serious side effects and can even address radiotherapy-induced leukopenia, alopecia areata [13, 14, 49], which indicates a good potential whether in single treatment or in combination therapy of cancers.

In conclusion, our present study demonstrates that CEP functions as the first β-catenin transcriptional inhibitor and effectively inhibits *APC*-mutant CRCs and suggests an attractive potential in the treatment of CRCs.

## Experimental section

### Reagents

CEP (Cepharanthine, purity > 98%, MB2077-S) was purchased from Meilunbio (Dalian, China). CHX (Cycloheximide, S7418), MG132 (S2619) and ActD (Actinomycin D, S8964) were obtained from Selleck (Houston, TX, USA). Promega (Madison, WI, USA) provided the product of dual-luciferase reporter assay kits (E1960). Invitrogen (Camarillo, CA, USA) supplied the product of Lipofectamine^®^ 3000 reagent (L3000015).

The antibodies used in the present investigation are as follows: β-catenin (Proteintech, 51067-2-AP), Axin2 (Abclonal, A17022), CyclinD1 (Proteintech, 60186-1-Ig), β-actin (Sigma-Aldrich, A1978), Lamin A/C (Proteintech, 10298-1-AP), Phospho-β-Catenin (Cell Signaling Technology, 9561), Active-β-Catenin (Cell Signaling Technology, 4270).

### Cell culture

The HEK293 cell lines originated from human embryonic kidneys; the SW480, SW620, and LoVo cell lines originated from human colorectal cancer; and the NCM460 cell lines originated from human normal colon epithelial cells. They were purchased through the Chinese Academy of Sciences. All the cells were cultivated following the manufacturer’s instructions.

### Plasmids

pGL3-β-catenin reporter plasmid was constructed by cloning the β-catenin promoter’s 2000 bp upstream region into the pGL3 plasmid (Promega). The Renilla luciferase plasmid was acquired from Promega. SuperTOPFlash plasmid was gift from Dr. Wei Wu (Tsinghua University, Beijing, China).

### Cell viability assay

The impact of CEP on cell survival was evaluated using an MTS reagent (Promega, G3581). 96-well plates were used to seed 5000 cells per well, which were then incubated overnight. Next, the cells underwent exposure to different amounts of CEP for 24 and 48 h. After removal of the culture medium, MTS solutions were introduced and cultured for one to four hours at 37 °C. The microplate reader (PerkinElmer) was applied to measure the optical density. Based on the dose–response curves, the IC50 values were derived and computed.

### Colony formation assay

Cell seeded were administrated with various doses of CEP for a period of 1–2 weeks. When clones were readily visible, they were washed in PBS and exposed to 4% paraformaldehyde for a duration of 15 min. The clones that were fixed were subsequently treated with 0.1% crystal violet for a duration of 30 min and then rinsed with distilled water. Finally, these stained clones were photographed and counted.

### Cell cycle analysis

Cells were seeded then treated with CEP for 24 h. Following the sample collection, the cell cycle analysis was conducted as previously [50]. The flow cytometer (BD Biosciences) was employed to measure the amount of DNA in the stained cells, and FlowJo software was utilized to analyze the results.

### Dual-luciferase reporter assay

96-well plates were employed to plant cells, which were then transfected with corresponding plasmids. After 6 h of transfection, cells were exposed to CEP for 24 h. Following this, the subsequent assay was conducted as previously [51]. The luciferase activity of fireflies was standardized by comparing it to the luciferase activity of Renilla.

### RNA interference

siRNAs targeting *CTNNB1* were produced by Qingke Biotechnology Company (Beijing, China). For *CTNNB1*, the siRNA sequences validated in the laboratory were used [51]. Lipofectamine 3000 transfected siRNAs for 48 h following manufacturer’s instructions.

### Western blotting assay

The collected cells, which had been subjected to either chemical treatment or siRNA transfection, got lysed with a potent RIPA buffer (Beyotime, Shanghai, China), including PMSF, and a cocktail of phosphatase inhibitors (Roche). Next, the supernatant extracts were measured using a BCA kit (Beyotime, P0009). Each of the protein extracts was divided into equal quantities and then uploaded to SDS-PAGE. After that, they were transferred to PVDF membranes (Millipore, ISEQ00010). Specific antibodies were exposed to membranes at 4 °C for an overnight period after blocking. Afterward, the membranes were exposed to suitable secondary antibodies under ambient temperature for a duration of 1 h. Eventually, an ImageQuant LAS 4000 mini (GE Healthcare) was used to visualize target protein bands that had been exposed to ECL substrate (Thermo Fisher, 32106). The grayscale of the indicated protein was quantified by ImageJ software.

### Immunofluorescence assay

96-well plates were used to seed 1.2 × 10^4^ cells per well, which were then exposed to various amounts of CEP for 48 h. Following the treatment, the subsequent immunofluorescence experiment was conducted as previously [51].

### Real-time PCR and RNA extraction

The Trizol reagent (Absin, abs60154), the reverse transcription kit (Takara, RR036A), and the SYBR reagents (Genestar, A301-10) were used for the extraction of RNA, subsequent reverse transcription, and finally real-time PCR, following the instructions provided by the respective manufacturers. Relevant primer sequences and data analysis methods were described previously [50].

### Cytoplasmic and nuclear protein extraction

To summarize, the cells were gathered and immersed in lysis buffer A. The mixture was then kept on ice for a duration of 10 min. Following centrifugation, the swollen cells were incubated for 2 min on ice in buffer A containing 0.2% NP-40. The liquid portion that represents the cytoplasmic fraction was obtained by spinning at 6000 rpm for 15 min. Following a rinse with lysis buffer A, the solid parts were suspended in lysis buffer B. Following a 15-min spin at 13,000 rpm, the liquid fraction was gathered as nuclear fraction. The procedure for preparing buffer A and B has been described in previous publications [51].

### Statistical analysis

To evaluate significance, two-tailed Student’s t-tests and two-way ANOVAs were used. This study used GraphPad Prism 8 to analyze the data. **p < 0.01; ***p < 0.001; *p < 0.05. P-values < 0.05 were significant.

## Data Availability

Upon reasonable request, data could be provided.
